# Context-specific variation and repeatability in behavioral traits of bent-wing bats

**DOI:** 10.1186/s40851-023-00206-9

**Published:** 2023-04-07

**Authors:** Yu-Jen Kuo, Ya-Fu Lee, Yen-Min Kuo, Yik Ling Tai

**Affiliations:** Department of Life Sciences, National Cheng Kung University, Tainan, 701 Taiwan

**Keywords:** Activity, Animal personality, Bats, Behavioral syndrome, Boldness, Exploration, Quadrupedal movement

## Abstract

Animals may show consistent among-individual behavioral differences over time and in different contexts, and these tendencies may be correlated to one another and emerge as behavioral syndromes. The cross-context variation in these behavioral tendencies, however, is rarely explored with animals in contexts associated with different locomotion modes. This study assessed the variation and repeatability in behavioral traits of bent-wing bats *Miniopterus fuliginosus* in southern Taiwan, and the effects of contextual settings associated with locomotion mode. The bats were sampled in the dry winter season, and their behaviors were measured in hole-board box (HB) and tunnel box (TB) tests, both suited for quadrupedal movements of the bats, and flight-tent (FT) tests that allowed for flying behaviors. The bats in the FT tests showed more interindividual and between-trial behavioral variation than those in the HB and TB tests. Nearly all of the behaviors in the TB and FT tests, but only half of those in the HB tests, showed medium to high repeatability. These repeatable behaviors were grouped into distinct behavioral traits of boldness, activity, and exploration, which were correlated to one another across contexts. In addition, we observed a consistently higher correlation between behavioral categories across the HB and TB contexts than between either of these contexts and the FT context. The results indicate consistent among-individual behavioral differences across time and contexts in wildly caught bent-wing bats. The findings of behavioral repeatability and cross-context correlations also indicate context-dependent variation and suggest that test devices which allow for flight behaviors, such as flight tents or cages, may provide a more suitable setting for measuring the behaviors and animal personalities of bats, particularly for those species that display less or little quadrupedal movements.

**Supplementary Information:**

The online version contains supplementary material available at 10.1186/s40851-023-00206-9.

## Background

Different tendencies in behavioral reactions or performances among individuals have long been noticed in both wild and captive animal populations. In former times, these differences were often treated as natural deviations from the adaptive mean, or just noise, and discounted [1]. With the rise of interest in animal personality, or sometimes referred to as temperament or coping style in different fields [2, 3], this trend has come under increasing challenge [4–6]. The notion of animal personality has widened the traditional view of animal behaviors and offered important insights and implications for the ecological and evolutionary mechanisms and fitness consequences of animal personality at different levels, from individual and population (e.g., [7–10]) to community (e.g., [11, 12]) and ecosystem services (e.g., [13]).

Previous studies on animal personality have identified certain behavioral traits that differ consistently among individuals (e.g., boldness and exploration) in diverse taxa [3, 14]. Many studies have also reported suites of correlated interindividual differences in behavioral types that occur consistently over time or situations and form behavioral syndromes [15, 16]. Along with birds and fishes, mammals have received considerable attention as subjects of animal personality studies [14, 17]. Most of these studies, however, have focused primarily on primates, rodents, and domestic or captive species that have generally been confined to farms, zoos, or laboratories [14–18].

Bats constitute the second largest mammalian order, next only to rodents, and account for over one-fifth of all mammals. Moreover, they display intricate behavioral features, along with morphological, physiological, and ecological adaptations. Many bats roost in colonies and seek shelter in various types of habitats, from caves, crevices, trees, and foliage, to human-made structures [19]. Bats in general are also gregarious mammals and engage in diverse social systems [20], wherein females and males often differ in dispersal potentials [21] and seasonal physiological needs due to asynchronous reproductive cycles [19]. Animal personality in bats, however, has not been studied to the extent reflecting their diversity. In fact, only a dozen or so related studies have been performed [22, 23], of which more than half have been devoted to just two species: the little brown bat, *Myotis lucifugus* [23–26] and the big brown bat, *Eptesicus fuscus* [22, 27].

With a few exceptions [28–31], the individual behavioral differences in bats have generally been assessed using hole-board boxes, mazes, or both [22–25, 27, 32]. Both devices are designed for testing behavioral tendencies mainly in terrestrial locomotion modes. Many vespertilionid bats, little and big brown bats included, are capable of quadrupedal movements to certain extents, particularly in roosting conditions (e.g., tree or rock crevices, tree hollows, and human-made structures such as roof cavities and nest boxes [19]). Nevertheless, most bats, other than perhaps *Desmodus rotundus* and *Mystacina tuberculate*, rely on sustained powered flight as the predominant locomotion mode due to morphological constraints, and some species even lack quadrupedal capability entirely (e.g., *Glossophaga* and *Macrotis* in phyllostomids [33]). Quadrupedal movement in most bats appears poorer than in other mammals [34], and is particularly strenuous for bats of long-narrow wingspans with relatively higher aspect ratios and wing loadings, which are adapted to aerial hawking in less cluttered open areas and long-distance flights (e.g., miniopterids, molossids [35]). Even for some fast aerial-hawking bats that are also agile crawlers, quadrupedal transport incurs considerably more cost than flying (e.g., *Molossus currentium* [36]). Yet, it is not known if the degree of consistent interindividual behavioral differences differ between the behaviors assessed by devices associated with different locomotion modes, i.e., flying vs. quadrupedal movement.

This study aimed to investigate whether consistent interindividual behavioral differences are detected in natural large bat aggregations. We assessed the tendencies and variation of eastern bent-wing bats, *Miniopterus fuliginosus*, in foraging and dispersal related behaviors (e.g., latencies, movement/locomotion activity, and exploration [3]), using different experimental devices mimicking various spatial conditions and contexts fitting with different locomotion potentials. We speculated that bats differ in behavioral types and predicted that differences in behavioral types among bat individuals are consistent over time and across contexts. Regarding the different experimental contexts, we tested whether the interindividual behavioral differences are correlated to one another across contexts and behaviors. We predicted a higher correlation between behaviors across contexts fitted with a similar locomotion mode than between those fitted with more dissimilar locomotion modes.

## Materials and methods

### Study animals and study area

Eastern bent-wing bats, *M. fuliginosus* (Miniopteridae), are widely distributed in East and Southeastern Asia. The field work was conducted in Guijijaou Experimental Forest (GEF; 20°58′N, 120°48′E; 200–300 m in elevation, ca. 450 ha in total area), Kenting, Taiwan. This area is characterized by a mean temperature of > 20 °C in January and an average temperature of > 28 °C in July–August. Moreover, the area has an annual rainfall of more than 2200 mm (Guijijaou Weather Station data, Taiwan Forestry Research Institute), with most of this rainfall occurring in the May–October monsoon season. The studied bats are common year-round residents in the GEF forest and are aggregated in several large colonies in coral reef karst caves [37].

### Bat sampling, treatments, and experimental preparation

Bat sampling took place from 2016 to 2018 between late fall and early spring. We sampled the bats once every 2–3 weeks at their dusk emergence using a custom-made net designed to cover only the lower portion of the cave exit. On every sampling occasion, we randomly sampled one or two bats every 3–5 min until 18–20 bats were captured. We measured the body mass of each bat using an electronic balance (JYB-500, Jin Yuan, Taiwan) and recorded the forearm length using a Vernier caliper (SV-03, E-Base, Taiwan). We distinguished the sex of each bat by the presence of genitalia and differentiated juveniles from adults by the gaped epiphyseal plate between the long phalanges. Juveniles and females showing any signs of reproductive stages, as determined by stomach palpation and the presence of nipple swelling and milk [38], were excluded from the behavioral tests and released on site.

The bats were brought to captivity and provided water *ad libitum* and mealworms at several intervals throughout the night, starting at dusk [39]. We measured the body mass of each bat to assess the body condition prior to each nightly behavioral test. After completing the test, the bats were placed individually in lidless containers (7 × 7 × 12 cm), each within a cloth bag for conserving humidity, and were hung in a cluster in a quiet dark room to spend the day. Drinking water was supplied individually to each container. We followed the guidelines in Reference [40] for handling and care of the bats throughout the study. All of the bats were released on the capture site after the experiments were completed.

### Behavioral experiments

The nightly behavioral tests were conducted over six consecutive days after settling the bats into captivity. We adopted three experimental devices: a hole-board box (HB), a tunnel box (TB), and a flight-tent (FT) tests, and alternated these three tests in each session. Each experimental setting aimed to reproduce specific real-world spatial situations or contexts in order to assess a wide suite of bat behaviors (the next sentence goes right after this sentence). In each setting, we randomly selected bats in a sequential testing order. Moreover, in each session, each test in the same setting was repeated after 48 h to evaluate the consistency of the bat behaviors [6, 41]. We acknowledge that this may not be a sufficiently long interval, given the practical difficulty of measuring the same bats repeatedly from a big colony over a longer period of time.

#### Hole-board box

**Fig. 1:**
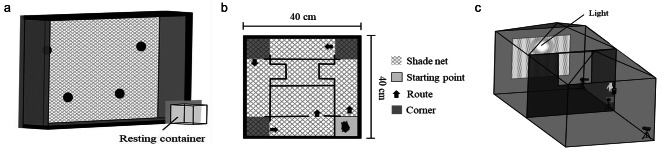
Experimental test devices: **(a)** hole-board box, **(b)** tunnel box, and **(c)** flight-tent

Hole-board boxes have been used to assess the activity, anxiety, and willingness to explore of small animals in open field tests, including rodents [42] and bats [24]. The hole-board box used in the present study had the form of a wooden box (60 × 40 × 10 cm) with a transparent acrylic lid placed over the top. We furnished a styrofoam board and black shade net (3 mm in mesh size) on the bottom base and along the four walls of the box to aid the bat with climbing. To evaluate the exploratory behavior of the bats, we also drilled four holes (2.5 cm in dia., 2 cm depth) into the base of the box (Fig. 1a), where two of these holes were set close to the center arena of the box (20 cm from either side of the box and 15 cm from the bottom edge) and the other two holes were positioned higher up and closer (8.5 cm) to both the top edge and the respective near-side edge of the box. The entrance and start line were set in the lower-right corner of the arena and were joined to an external resting container (10 × 8.5 × 11.5 cm). The box was set vertically to stimulate the climbing behavior of the bats, and each bat was tested individually for 10 min. In each test, the bat was placed in the resting container to settle for 60 s and the sliding door to the arena entrance was then opened. We allowed 60 s for the bat to enter the arena voluntarily like an emergence test [41]; or else we gently nudged the resting container into the arena. We then closed the sliding door and observed the bat behavior for 10 min. On completion of the test, we cleaned the arena and resting container using paper napkins and water in order to remove any trace of animal scent or marks.

#### Tunnel box

The tunnel box had the form of a wooden box (40 × 40 × 12 cm) and generally resembled the hole-board box in its exterior framework and side layout, with an exception of the center arena (Fig. 1b). The inside of the box was designed to imitate the maze-like rugged wall surface of a karst cave and comprised four tunnels (40 × 6 × 12 cm), four turning corners (8 × 8 × 12 cm), and one center arena (24 × 12.5 × 12 cm). A cardboard box (6 × 6 × 12 cm) was inserted into each of the left and right tunnels as a refuge. In addition, a resting container (8 × 8 × 12 cm) and a sliding door were positioned at the lower-right corner of the box as the starting point. As in the hole-board box tests, the tunnel box was set vertically during the tests. Prior to each test, the bat was placed in the resting container for 60 s to settle and the sliding door was then opened like an emergence test [41]. Once the bat entered the tunnel box, either voluntarily or by gently pushing the resting container into the arena, its behavior was continuously observed for 10 min. On completion of the test, the box and resting container were also cleaned as described for the hole-board box.

#### Flight-tent

A flying space (640 × 320 × 320 cm) was constructed outdoors on a grassy area and under a tree canopy by joining two identical tents (Big Lion TURBO320, Taiwan) side by side (Fig. 1c). A black curtain was placed between the tents with a rectangular window (85 × 75 cm) cut into it to serve as a passageway between the two spaces. The mesh window on the end side of the back tent allowed the bats’ call signals to pass through. We fixed a D230 bat detector (Pettersson Elektronik AB, Uppsala, Sweden) at the side of the front tent facing the start point to record the latency and duration of echolocation calls of the bats [28]. We recorded the bats’ flight behaviors using three video cameras (SONY 4 K FDR-AX55, Japan) strategically positioned in the tents and aided by an infrared lamp hung from the top of the first tent. Video recording commenced immediately when the bat was positioned at the start point until 2 min after the bat took off, or a total of 10 min elapsed.

#### Behavioral assessments

In each setting, we operated the video cameras in the night-shot mode to record the bat behaviors, aided by an infrared lamp. We measured multiple behaviors in all of the three tests [41]. In the HB tests, we timed the emergence latencies from the resting container, the time spent climbing and grooming, and the number of head-dipping at the holes [24]. In the TB tests, we also timed the emergence latencies from the resting container and from each turning of a corner [23], the time spent climbing and grooming, and the number of refuge-visits. Finally, in the FT tests, we measured the call latency and duration of echolocation [28], the departure latency, the latency and the number of passes through the passageway, the number of circlings of the tent, and the total time in flight and spent grooming.

To measure the bat movements in the HB and TB boxes, we used VLC Media Player 3.0.11 (Paris, France) to capture bat trajectories and then used NIH Image J 1.52 (Bethesda, MD, USA) to measure the distance (cm) and area (cm^2^) climbed by the bats based on the reference marks with a scale of 0.1 cm. We further calculated the climbing speed and efficiency using the distance and area climbed divided by the time spent moving (sec), respectively. The percent flying time in the FT tests was evaluated by dividing the time spent flying by the total testing time (120 s). In addition, the circling rate was calculated as the total number of circlings divided by the total testing time. Finally, we calculated the pass rate by dividing the total number of passes through the passageway by the testing time.

Some behaviors may reflect similar specific functional tasks (e.g., locomotion activity, information gathering) or emotional state (e.g., latency to initiation), whereas their expression may be affected to different extents across various test contexts due to locomotive constraints or intermingled influences. Thus, we adopted existing animal personality terms to categorize the traits we observed according to frequently used definitions [3]. Boldness is response to potential risky situation, which included the latencies of the bats emerging from the resting container [43, 44], together with those from the TB entrance, start-point in the flight-tent, and window passing, respectively. Activity behaviors refer to the presence or absence of movement, its intensity, or the general level of locomotion activity, whereas exploration typically refers to the reaction to a new (i.e., novel) situation aiming for information gathering [3]. These two behavioral traits may appear similar but may be affected differently by different underlying motivations or other constraints, for instance when tested in a limited space or in a space with limited movement potentials. Presumably this limitation would also affect bats tested in different situations associated with different locomotion modes. Thus, the percent time spent climbing in a box [24] or flying in the tents, the distance and area climbed in the center arena, and the total number of circlings and window passings in the flight-tent were treated as measures of activity. Finally, the speed and efficiency of climbing, circling rate, pass rate, number of head-dippings in the HB tests, number of recess-visits in the TB tests, and echolocating call duration in the FT tests were taken as measures of exploration [32, 45, 46].

### Data analyses

Data are presented as the mean ± standard error (*SE*) or relative proportion (%), unless otherwise noted. We conducted all of the statistical tests with an alpha value of 0.05 using SPSS for Windows (28.0, IBM Chicago, Illinois, USA). We performed arcsine, square-root, and log (*x* + 1) transformation for proportional, count, and time data, respectively, as necessary, to meet the normality. The behavioral tendencies of the bats between sex and repeated trials were examined using multivariate analysis of variance (MANOVA) tests followed by post hoc analyses using Fisher’s LSD to locate the differences [47].

In assessing the consistency of the bat behaviors, we retained the behaviors performed by at least half of the bats for analyses and evaluated the repeatability (*R*) of the behaviors in the two repeated trials via the proportion of the total variance accounted for by the interindividual differences, i.e., the intra-class coefficient correlation (ICC; [48]). We applied the generalized linear mixed model (GLMM) with the restricted maximum-likelihood method, using R package (v 3.6.0, R Core Team 2019), to estimate the repeatability under consideration of both the within- and among-individual variances. In accordance with the criteria of Bohn et al. [49], we defined *R* values < 0.2 as weak, ≥ 0.2 but ≤ 0.4 as medium, and > 0.4 as strong [6]. We considered behaviors with *R* values ≥ 0.2 as consistent behaviors over time and further classified these consistent behaviors into one of the behavioral categories as defined above. If any behavioral category contained two behaviors or more, we used principal component analysis (PCA) with varimax rotation on the repeated measures of each behavior to reduce the behavioral variables to fewer components [50]. We followed the Kaiser-Guttman criterion and accepted components with eigenvalues > 1 as the dominant variables of the corresponding behavioral traits [51]. We calculated the correlations of the resultant behavioral traits in PCA scores among different test contexts using the Pearson’s correlation coefficient (*r*) [41].

## Results

### Behavioral tendencies, variation, and their repeatability

We tested a total of 218 bats over 15 sessions. In the hole-board (HB) tests, the bats showed a between-sex difference (MANOVA, Pillai’s *V* = 0.06, *F*_8, 422_ = 3.09, *p* < 0.01), with the male bats climbing over a larger area (749.29 ± 55.88 cm^2^) than the females (538.72 ± 43.05 cm^2^; Fisher’s LSD *p* < 0.005). The bat behaviors, however, did not differ between the two trials (*V* = 0.02, *F*_8, 422_ = 1.24, *p* = 0.28; sex × trial interaction *p* > 0.32). The climbing speed and emergence latency showed medium repeatability (*R* = 0.24–0.27), while high repeatability occurred only in the area climbed (*R* = 0.54). The other behaviors all showed low repeatability (*R* < 0.2; Table 1).

**Table 1:** Mean (± *SE*) behavioral performances and behavioral repeatability (assessed by *R* value with 95% CI in parenthesis) of eastern bent-wing bats in hole-board box tests.

Behavioral measurements	Trial 1 (*n* = 217)	Trial 2 (*n* = 216)	Repeatability
Emergence latency	249.22 ± 14.90	214.97 ± 14.52	**0.24** (0.11, 0.35)
Percent time climbing	14.92 ± 1.05	14.56 ± 1.05	0.12 (0, 0.24)
Distance climbed	199.13 ± 17.74	199.57 ± 25.31	0.18 (0.05, 0.31)
Area climbed^1**^	604.89 ± 47.72	649.63 ± 50.23	**0.54** (0.44, 0.62)
Climbing speed	1.77 ± 0.10	1.90 ± 0.11	**0.27** (0.14, 0.39)
Climbing efficiency	1.99 ± 0.12	1.93 ± 0.12	0.11 (0, 0.24)
Head-dipping	0.04 ± 0.02	0.07 ± 0.02	---^2^
Grooming	21.14 ± 3.48	15.85 ± 3.20	---^2^

^1^Between-sex difference, ^**^p < 0.01; ^2^ Excluded from the repeatability analysis because the behavior occurred in less than half of the bats tested.

The bat behaviors in the tunnel box (TB) tests differed between the two trials (*V* = 0.08, *F*_9, 417_ = 4.08, *p* < 0.001), but not between sex (*V* = 0.02, *F*_9, 417_ = 0.78, *p* > 0.63; sex × trial interaction *p* > 0.68). The bats emerged earlier from the resting container, climbed over a larger area, visited the refuge boxes more frequently, and spent less time grooming in the second trial (Fisher’s LSD, all *p* values < 0.05, Table 2) than in the first trial. All of the behaviors showed medium repeatability (*R* = 0.21–0.39), other than the climbing efficiency, which showed a high repeatability (*R* = 0.44, Table 2).

**Table 2:** Mean (± *SE*) behavioral performances and behavioral repeatability (assessed by *R* value with 95% CI in parenthesis) of eastern bent-wing bats in tunnel-box tests.

Behavioral measurements	Trial 1 (*n* = 215)	Trial 2 (*n* = 214)	Repeatability
Emergence latency^1*^	232.22 ± 15.67	177.26 ± 15.21	**0.21** (0.07, 0.33)
Corner emergence latency	453.01 ± 11.10	421.70 ± 12.01	**0.33** (0.20, 0.45)
Percent time climbing	1.36 ± 0.11	1.62 ± 0.12	**0.31** (0.18, 0.42)
Distance climbed	106.23 ± 8.54	112.00 ± 7.70	**0.33** (0.21, 0.44)
Area climbed^1**^	377.13 ± 17.78	460.72 ± 21.81	**0.35** (0.21, 0.46)
Climbing speed	0.71 ± 0.06	0.80 ± 0.05	**0.39** (0.26, 0.50)
Climbing efficiency	3.36 ± 1.31	1.88 ± 0.70	**0.44** (0.33, 0.54)
Recess-visiting^1*^	0.22 ± 0.04	0.42 ± 0.07	---^2^
Grooming^1*^	39.18 ± 4.87	23.42 ± 4.05	---^2^

^1^Between-trial difference, ^*^p < 0.05, ^**^p < 0.01; ^2^ Excluded from the repeatability analysis because the behavior occurred in less than half of the bats tested.

In contrast to the HB and TB tests, the behaviors in the flight-tent (FT) tests differed both between trials (*V* = 0.13, *F*_10, 400_ = 6.18, *p* < 0.001) and between sexes (*V* = 0.07, *F*_10, 400_ = 3.18, *p* < 0.001; sex × trial interaction *p* > 0.47). In particular, the bats departed from the start point earlier and spent less time calling before departure, but spent a longer passageway latency, in the second trial (Table 3). Moreover, the males departed (164.17 ± 12.47 s) and emitted calls (24.92 ± 4.40 s) more quickly than the females (departure: 217.59 ± 16.57 s; call: 41.19 ± 7.15 s), and had a greater total number of passes (3.0 ± 0.34), circle rate (0.36 ± 0.010), and pass rate (0.03 ± 0.003) than the females (pass: 1.7 ± 0.21; circle rate: 0.33 ± 0.013; pass rate: 0.02 ± 0.002). All of the behaviors showed a high repeatability (*R* = 0.58–0.69), with the exception of the pass latency (*R* = 0.38) and call duration (*R* = 0.29) with medium repeatability, and call latency with low repeatability (Table 3).

**Table 3:** Mean (± *SE*) behavioral performances and behavioral repeatability (assessed by *R* value with 95% CI in parenthesis) of eastern bent-wing bats in flight-tent tests.

Behavioral measurements	Trial 1 (*n* = 215)	Trial 2 (*n* = 214)	Repeatability
Departure latency^1***, 2**^	237.61 ± 14.56	153.65 ± 13.63	**0.67** (0.58, 0.74)
Pass latency^1***^	60.78 ± 3.68	76.73 ± 3.44	**0.38** (0.26, 0.49)
Percent time flying	70.69 ± 2.76	76.09 ± 2.63	**0.58** (0.48, 0.66)
Total circling	35.19 ± 1.42	38.56 ± 1.39	**0.64** (0.56, 0.71)
Total pass^2**^	2.65 ± 0.28	2.24 ± 0.31	**0.59** (0.50, 0.68)
Circling rate^2*^	0.33 ± 0.01	0.36 ± 0.01	**0.69** (0.62, 0.76)
Pass rate^2***^	0.03 ± 0.003	0.02 ± 0.003	**0.64** (0.55, 0.72)
Call latency^2*^	28.15 ± 6.67	35.50 ± 4.51	0.18 (0.05, 0.31)
Call duration^1**^	44.75 ± 3.91	26.60 ± 3.64	**0.29** (0.16, 0.41)
Grooming^1^	4.39 ± 2.00	1.74 ± 1.03	---^3^

^1^Between-trial and ^2^between-sex differences, ^*^p < 0.05, ^**^p < 0.01, ^***^p < 0.001; ^3^Excluded from the repeatability analysis because the behavior occurred in less than half of the bats tested.

### Behavioral traits and among-trait correlations

Each of the three consistent behavioral measurements in the HB tests corresponded to a single behavioral category, i.e., emergence latency for boldness, area climbed for activity, and exploring efficiency for exploration. In contrast, the behavioral measurements with medium and high repeatability in the TB tests were loaded onto a respective PCA axis under each behavioral category, and each component explained 66.98–80.36% of the total variance of the corresponding category (Table 4). In the FT tests, the behaviors of medium and high repeatability were also loaded onto a respective PCA axis under each category, and each explained 51.64–73.21% of the total variance of the corresponding trait, respectively (Table 4).

**Table 4:** Behaviors with medium or high repeatability (*R ≥ 2*) displayed by eastern bent-wing bats in tunnel-box and flight-tent tests, and the respective PC loadings, cumulative variance explained, and eigenvalues (*λ*) shown under each behavioral category.

Behavior/setting	Tunnel-box	Flight-tent
**Boldness**		
Emergence/departure latency	0.90	0.86
Corner emergence latency	0.90	
Pass latency		0.86
Variance (%)	80.36	73.21
*λ*	1.61	1.46
**Activity**		
% time climbing/flying	0.95	0.95
Distance climbed	0.98	
Area climbed	0.96	
Total circling		0.97
Total pass		0.65
Variance (%)	92.40	75.60
*λ*	2.77	2.27
**Exploration**		
Climbing speed	0.82	
Climbing efficiency	0.82	
Circling rate		0.86
Pass rate		0.61
Call duration		0.67
Variance (%)	66.98	51.64
*λ*	1.34	1.55

**Fig. 2:**
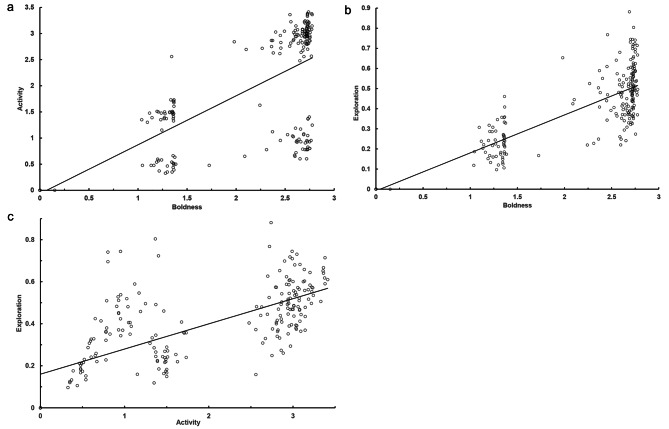
Correlation of normalized values of repeatable behaviors between paired behavioral categories **(a)** boldness-activity, **(b)** boldness-exploration, and **(c)** activity-exploration assessed for eastern bent-wing bats in hole-board box tests

**Fig. 3:**
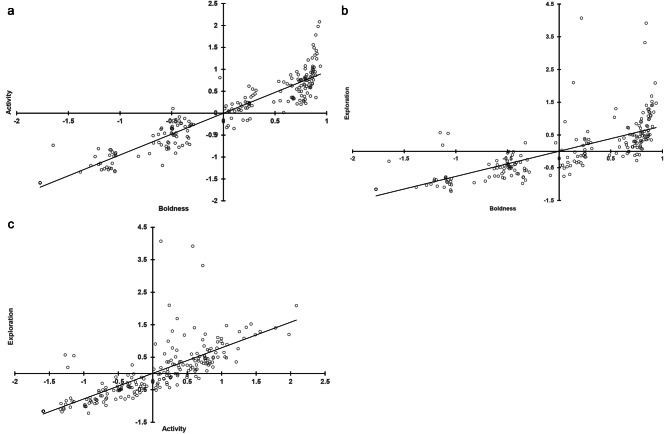
Correlation of PC scores between paired behavioral categories **(a)** boldness-activity, **(b)** boldness-exploration, and **(c)** activity-exploration assessed for eastern bent-wing bats in tunnel box tests

**Fig. 4:**
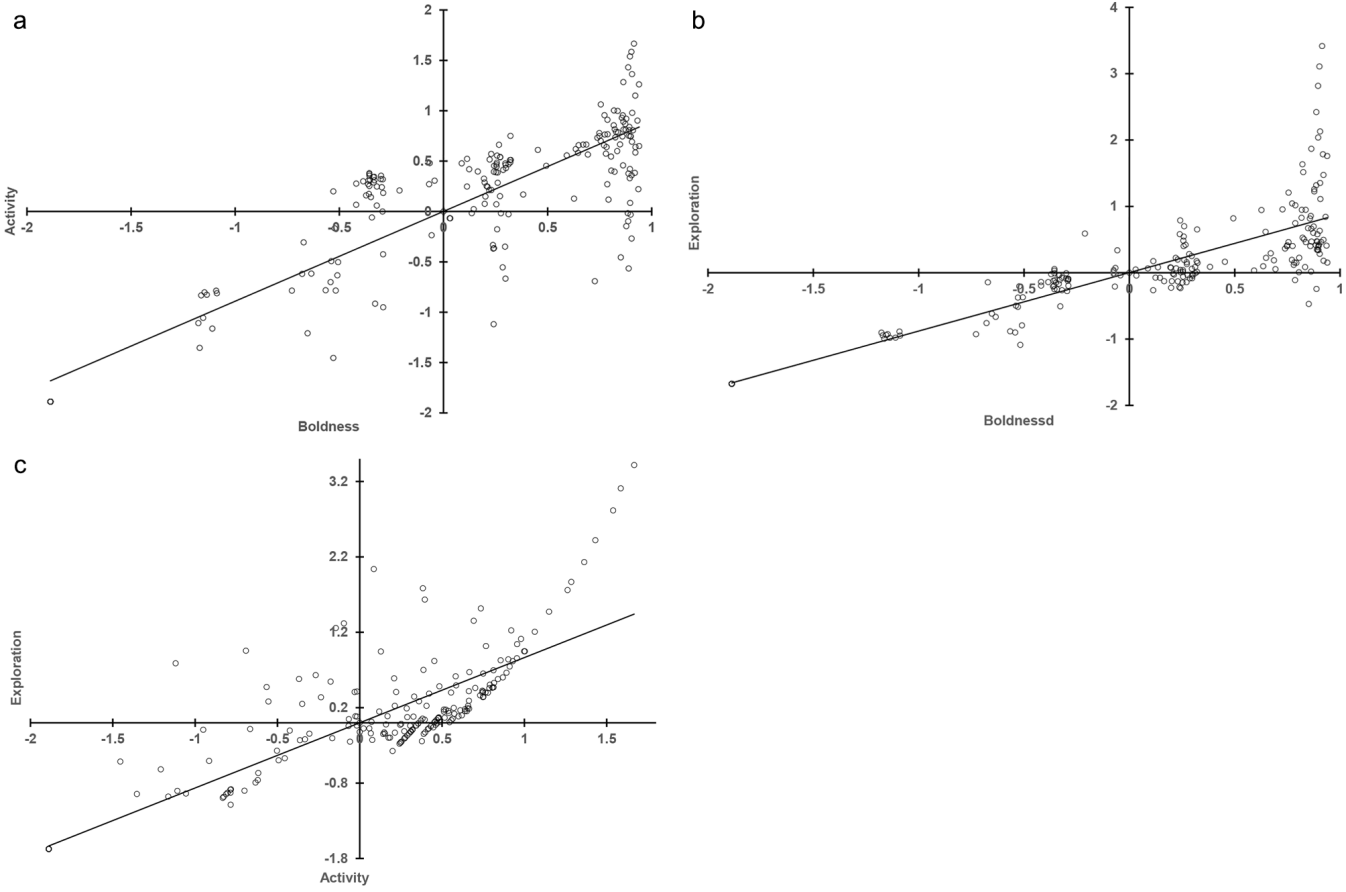
Correlation of PC scores between paired behavioral categories **(a)** boldness-activity, **(b)** boldness-exploration, and **(c)** activity-exploration assessed for eastern bent-wing bats in flight-tent tests

Between-context, the HB and TB tests showed medium correlations in PC scores for all three behavioral categories (*r* = 0.35–0.48, all *p* values < 0.05; Table 5), but only lower correlations were detected between the HB and FT tests (*r* = 0.31–0.32, all *p* values < 0.05), whereas those between the TB and FT tests were mostly weak or negligible (*r* < 0.25, Table 5). Within each context, the three behavioral categories of the bats were positively correlated to each other. The boldness and activity tendencies of the bats appeared bimodal in the HB tests (boldness-activity: *r* = 0.71, boldness-exploration: *r* = 0.82, activity-exploration: *r* = 0.69; Fig. 2), but less, or not so, in the TB tests (boldness-activity: *r* = 0.94, boldness-exploration: *r* = 0.73, activity-exploration: *r* = 0.76; Fig. 3) and the FT tests (boldness-activity: *r* = 0.89, boldness-exploration: *r* = 0.86, activity-exploration: *r* = 0.87; Fig. 4). By contrast, the exploration tendencies of the bats were distributed more continuously among individuals.

**Table 5:** Correlations of different behavioral categories in PCA scores across different test contexts. Boldness, activity, and exploration assessed across hole-board box (HB) and tunnel-box (TB) tests were consistently more correlated to one another (*r* > 0.35) than between flight-tent (FT) tests with either HB or TB tests (*r* < 0.35 or lower)

Behavior	Boldness		Activity		Exploration	
	HB	TB	HB	TB	HB	TB
Boldness						
TB	**0.479**		**0.361**		**0.426**	
FT	0.205	0.231	**0.316**	0.227	**0.308**	0.125
Activity						
TB	**0.450**		**0.396**		**0.434**	
FT	0.180	0.157	0.261	0.168	**0.314**	0.080
Exploration						
TB	**0.383**		**0.354**		**0.374**	
FT	0.211	0.171	**0.324**	0.173	0.292	0.108

## Discussion

This study presents the first description of animal personality in widely caught bent-wing bats and in East Asian tropical forests. The traits we identified as displaying consistent individual differences included behaviors associated with exploration and activity, which have been reported in temperate *M. lucifugus* [24, 25] and *E. fuscus* [22, 27], as well as in several other bats [31, 32, 52]. Our data, however, also uncovered an additional behavioral aspect regarding risk-taking tendency, namely boldness, which has thus far been described only rarely in bats [31]. Our study assessed multiple behaviors using multiple experimental devices imitating different spatial contexts that may be encountered by the bats in natural situations. As predicted, these devices revealed different behavioral variations of the bats.

We found a sex difference in the behavioral tendencies of the bats, but not in all behaviors, nor in all test settings. The males showed shorter departure and call latencies, and a greater number of passes and higher circling and pass rates in the FT tests, and a larger area climbed in the HB tests, than the females. These differences suggest a tendency directed toward a more proactive male behavioral type, and concur with the male-biased dispersal reported for bats in general [21], and specifically for the closely related common bent-wing bat *Miniopterus schreibersii* [53]. Sex differences in animal behaviors have also been reported in other studies (e.g., [54–56]; also reviews in [57]). Across-species patterns in sex-dependent animal personality, however, appear insignificant or inconclusive (e.g., [58, 59]), which suggests that sex differences in behavioral type may be subject to species and what or how the behaviors of the selected species are measured [60]. For instance, the analyses of Harrison et al. [59] included only one bat species from a single study considering a single trait, and little is known about the individual differences and between-sex behavioral tendencies in most bats.

Consistent behavioral differences among individuals do not necessarily mean fixed behaviors [61]. In fact, some behavioral tendencies of the present bats changed across the two trials. This may be due to a habituating effect to the testing context, which can occur in repeated behavioral trials (e.g., [62, 63]). However, the reduced latencies observed in the second trials in the TB and the FT tests, respectively, may also stem from a prolonged response time to a novel environment in the first encounter [64]. Furthermore, the fact that the bats spent a longer time emitting echolocating calls in the first trial in the FT tests suggests a process of information collection aimed at assessing potential risks. That is, bats may initially use calls to familiarize themselves with a novel situation (e.g., *M. lucifugus*, [65]), and then gradually reduce the frequency and duration of calls as they become more acclimated to the flight space in which they find themselves (*E. fuscus*, [66]). A similar acoustic exploration behavior is also observed in *Pipistrellus nathusii* [32], whereas the contact calls of Spix’s disk-winged bats, *Thyroptera tricolor*, revealed consistent individual differences associated with different social contexts [28]. In general, the results suggest that echolocation calls are a potentially important yet still relatively unexplored avenue in the study of the behavioral types of bats.

Given the individual variation in some of the behaviors exhibited by the present bats over time, the interindividual differences in behavioral measurements were largely consistent and repeatable across the three contexts. The between-test interval in our study was not sufficiently long for notable changes in individual state [6], so the results may represent only short-term repeatability [67, 68]. Nevertheless, the behavioral repeatability results in our study are generally comparable to those of previous studies on the personality of bats (*r* = 0.13–0.69; [22–24, 30]) and the averaged repeatability (*r* = 0.37) of behavioral measures over 98 species of animals, ranging from insects, to fish, reptiles, birds, and mammals [6]. Some of the behaviors in our study, however, showed a lower repeatability (*R* < 0.2), most notably in the HB tests. A lower repeatability in behavioral tendencies may be the result of unpredictable individual variability or a shifting in responses over time (e.g., habituation or sensitization), both of which can cause behavioral convergence, or the reverse [69–71]. Our present study design does not permit assessing the effects of habituation, which presumably would be greater in the HB tests where the smaller, more confined but less complex test arena is easier to investigate by bats using echolocation. Indeed, habituation has been observed in open-field tests of various taxa, including those applying the hole-board device [69].

In the present study, the low repeatability may also result from the differences among the three testing devices and the corresponding changes induced in the associated contexts. Among the three contexts, the TB context was most confined in space of the three settings, but nevertheless resembled the real-life situation in which the bats climb along rugged wall surfaces while inside their roost in a karst reef cave. The HB setting was less confined than the TB setting, and the center arena of the box served as a novel open area that potentially exposed the bats to a riskier situation [72]. In comparison, the FT context represented an even more open space than the TB or HB setting to the bats; however, it also allowed the bats to engage in their predominant and natural locomotion mode, namely, powered flight.

Flying significantly increases the ability of bats to avoid and evade potential risks [73]; thus, being able to fly can reduce their vulnerability as a result. The fact that flying was much more restricted or completely inhibited in the HB and TB settings may have caused the bats to incur increased vulnerability in a risky situation, particularly in the HB setting. This effect presumably may have created a difference in the situational strength associated with the different test devices, and the effects of the situation on individuals’ behaviors [41]. For instance, flight attempts (i.e., wing-flapping) were recorded in *M. lucifugus* [24], but we observed no such behavior and rarely head-dipping in the present study. This may further explain the observed bimodal distribution of the PC scores of the boldness and activity traits in the HB tests. Other potential biases may also arise from the floor or ceiling effects (e.g., cutting off data, [61, 69]) since the test settings encouraged prolonged and inhibited behavioral responses or performance.

The low to medium positive correlations between different behavioral traits across the three contexts suggest the presence of a continuum of proactive-reactive behavioral response [2] for the tested bats, wherein the bats responding more proactively tended to be bolder, more active, and also more exploratory. Our analysis revealed only phenotypic correlations without partitioning the residual component from the between-individual correlation [67], which although it is likely valuable [78], is found reasonably well described by phenotypic correlation [17, 74].

A behavioral syndrome among the aspects of exploration, activity, and sociality has been previously reported in *M. lucifugus* [23]. In Asian particolored bats, *Vespertilio sinensis*, however, no evidence of such a syndrome was found among the aspects of exploration, activity, and aggression for female bats [52]. Furthermore, behavioral syndromes may not exist in all contexts for the same species either (e.g., [75, 76]), which indicates a context-specific nature of the behavioral variation [16]. Our findings of a difference in the correlations between paired contexts also suggest that the behavioral traits of bats may be context-specific. This difference concurs with previous findings of a lower correlation between exploration and activity within different contexts and the presence of context-specific behavioral traits in animal personality of great tits [77].

The lower correlations observed in the behavioral traits between the FT setting and either the HB setting or the TB setting again imply a potential link between the behavioral assays conducted and the different locomotive patterns available to the bats. Bats face very different ecological conditions while flying in the air than when performing quadrupedal climbing or crawling along a substrate (e.g., caves), and the different environmental stresses may in turn affect the behavioral performance associated with different behavioral types. Bats whether flying in the air or roosting in caves may be exposed to potential risks of predation, although of different kinds and extents [73]. Nevertheless, flight plays a predominant role in the locomotion modes available to bats, and is always their easiest and quickest way of evading a threat [19]. The natural flight behavior of bats, however, cannot be fully accessed in the current form of a typical hole-board box.

## Conclusions

Our results indicate the existence of consistent behavioral differences over time and across contextual settings in wildly caught eastern bent-wing bats, wherein bolder bats also tended to be more active and more exploratory. Moreover, the findings of behavioral repeatability and cross-context correlations suggest that, for further assessing the personality or behavioral plasticity of bats, experimental devices which allow for flight behaviors, such as flight-tents or cages, may provide a more suitable and realistic setting for flight-related behaviors and situations, particularly for bats that display less or little quadrupedal movements (e.g., species in Hipposideridae, Rhinolophidae, Mormoopidae, Natalidae, and Phyllostomidae; [33, 78]). The size of the test arena needs to be considered to better incorporate species differences in flight modes and speed ranges resulting from the different wing shape and morphology of bats [35]. The proper complexity in interior layout is essential, so habituation to the setting is not too quickly built, to better discriminate individual difference in responses [6].

## Electronic supplementary material

Below is the link to the electronic supplementary material.

Supplementary Material 1

Supplementary Material 2

## Data Availability

Please contact the corresponding author.
