# Supplementary material for: Context-specific variation and repeatability in behavioral traits of bent-wing bats
Source: Zoological Lett. 2023 Apr 7;9:8. doi: 10.1186/s40851-023-00206-9 (PMC10080966; doi:10.1186/s40851-023-00206-9)
Supplement: Supplementary file 1 — Supplementary Material 1 [file 40851_2023_206_MOESM1_ESM.doc]

20 January, 2023

Editors-in-Chief

Shigeru Kuratani, Evolutionary Morphology Laboratory, RIEN, Japan

Dear Prof. Kuratani:

Attached please find a secondarily revised version of the manuscript initially entitled “**Context-specific behavioral variation and repeatability in personality traits of bent-wing bats**” (ZLET-D-22-00030) by Kuo et al. It had been previously reviewed and given a decision of revision.

We are very grateful for the reviewers’ valuable comments and the opportunity offered to revise our manuscript. We have checked all the reviewers’ comments and conducted revisions accordingly, or provided further explanations. The revision includes revising the use of terminology and associated contents throughout the text, figure legends, and table captions, adding more information while removing some ambiguous or confusing words to improve clarity in different parts of the text, including the Methods and Discussion sections. In addition, the title of this manuscript has also been slightly revised like that appeared in the previous revision as “**Context-specific variation and repeatability in behavioral traits of bent-wing bats”.** We are now submitting this revision to Zoological Letters. The complete copy of point-to-point responses is attached with the online submission.

This manuscript is being submitted exclusively to Zoological Letters, and no part of this paper has been published, or is being considered for publication, elsewhere. We confirm that our study adhered to the ethical guidelines of the journal, and specifically provided the information of the funding source with grant-approval number in the Acknowledgments. We also declare no competing interests.

Thank you in advance for your time and effort regarding the enclosed materials.

Sincerely,

Ya-Fu Lee, Ph.D., Professor

Department of Life Sciences

National Cheng Kung University

Tainan 701, Taiwan

Tel: 886-6-2757575 ext. 58115
