# Supplementary material for: Context-specific variation and repeatability in behavioral traits of bent-wing bats
Source: Zoological Lett. 2023 Apr 7;9:8. doi: 10.1186/s40851-023-00206-9 (PMC10080966; doi:10.1186/s40851-023-00206-9)
Supplement: Supplementary file 2 — Supplementary Material 2 [file 40851_2023_206_MOESM2_ESM.doc]

We are very grateful for the further valuable comments, suggestions, and detailed editing advises for our manuscript. We have made revisions and provided justifications accordingly. The revisions included rephrasing the use of terminology and associated contents throughout the text, figure legends, and table captions, and adding more explanations or justifications while removing some ambiguous or confusing words or sentences to improve clarity in different parts of the text, including in the method and discussion sections. Our point-to-point responses to all comments are as follows. The responses are arranged by the same sequential order of the comments as that provided in the original comment sheets from the reviewers, and are line-numbered according to the line numbers initially present in the previous version and mentioned by the reviewers, unless otherwise noted. The new line numbers associated with the revisions are provided in parentheses.

**Reviewer 1.**

We are very grateful for the encouraging and positive comments, and many thoughtful suggestions and comments for revisions, particularly regarding to the proper use of terminology and the treatments on behavioral syndromes.

**General comments**

**1.** We excluded the across-context correlations of the same behavioral trait because we set no prior assumption of the focus of the study on the tests or the traits, whereas a comparison of correlations of the same traits across contexts might mislead toward the effect of test assay alone. We did calculate the across-context correlations of the same behavioral trait and found no differences from the pattern revealed from the correlations of different traits across different test contexts.

Thanks for the reminding comment and we have added those correlations back to the table presentation to show the full range of correlations of behavioral categories across contexts (**Table 5 in the revision**).

**2.** Thanks for the comment, and we have added a brief discussion on this issue as “…The between-test interval in our study was not sufficiently long for notable changes in individual state [6], so the results may represent only short-term repeatability [67, 68]” (**Lines 347-349 in the revision**). We also have addressed the issue of habituation and revised the relevant part of the paragraph as “…A lower repeatability in behavioral tendencies may be the result of unpredictable individual variability or a shifting in responses over time (e.g., habituation or sensitization), both of which can cause behavioral convergence, or the reverse [69-71]. Our present study design doesn’t permit assessing the effects of habituation, which presumably would be greater in the HB tests where the smaller, more confined but less complex test arena is easier to investigate by bats using echolocation. Indeed, habituation has been observed in open-field tests of various taxa, including those applying the hole-board devise [69].” (**Lines 354-360 in the revision**).

**3.** Thanks for the reviewer’s comment. Our results and conclusion convey no intention to suggest abandoning the use of hole-board box in studying animal personality of bats. There are certainly contexts where the hole-board box is suitable as a test array, such as sociability and mating behaviors that happen in roosting environments where quadrupedal movements are more often likely involved. Instead, our results indicate a need to properly choose suitable assays to better reveal the full range of variation in behaviors of interest. We also have provided some suggestive guidelines as **“**The size of test arena needs to be considered to better incorporate species differences in flight modes and speed ranges resulted from the different wing shape and morphology of bats [35]. The proper complexity in interior layout is essential, so habituation to the setting is not too quickly built and to better discriminate individual difference in responses [6].” (**Lines 418-421 in the revision**).

**Minor comments**

**Line 33.** We have changed the words to “…behavioral syndromes” (**Line 33 in the revision**)

**Line 42.** We have replaced “loaded” with “grouped” (**Line 42 in the revision**)

**Line 44.** The revision has rephrased the words as “…a consistently higher correlation between behavioral categories across the HB and TB contexts…” (**Lines 44-45 in the revision**)

**Line 65.** The revision has rephrased the sentence as “…have identified certain behavioral traits that differ consistently among individuals (e.g., boldness and exploration) in diverse taxa” as suggested (**Lines 65-66 in the revision**)

**Line 78.** We have added “s” to correct the word as “cycles” (**Line 79 in the revision**)

**Line 79.** We have rephrased the sentence as “Animal personality in bats, however, has not been studied to the extent in reflecting their diversity.” (**Lines 79-80 in the revision**)

**Line 97-98.** The revision has rephrased the sentence as “…if the degree of consistent interindividual behavioral differences differ…” (**Lines 98-99 in the revision**)

**Line 205.** The revision has changed the words to “…measure the distance (cm) and area (cm^2^) climbed by the bats…” (**Line 208 in the revision**)

**Line 216.** The sentence has been revised as “Thus, we adopted existing animal personality terms to categorize the traits we observed according to frequently used definitions...” as suggested (**Lines 218-219 in the revision**)

**Line 217-224.** Thanks for the comments and we have revised this part of the paragraph by incorporating the suggestion of the reviewer (please see **Lines 219-233 in the revision**). Specifically, we have added a brief explanation why we would differentiate activity from exploration as “These two behavioral traits may appear similar but may be affected differently by different underlying motivations or other constraints, for instance when tested in a limited space and movement potentials. Presumably this limitation would also affect bats tested in different situations associated with different locomotion modes” (**Lines 225-228 in the revision**)

**Line 242.** We have rephrased the words as “…into one of the behavioral categories as defined above” (**Line 251 in the revision**)

**Line 243.** Yes, we used the repeated measures of each bat in conducting the PCAs for the behavioral traits, and we have added this information in the revision (**Line 253 in the revision**)

**Line 278.** We have corrected the word as “measurements” (**Line 287 in the revision**)

**Line 281.** The revision has replaced “respective PCA axis” with “a respective PCA axis” (**Line 290 in the revision**)

**Lines 279, 282, 284, 287, 290, 607, 610, 614, 739, 770.** We have replaced “behavioral traits” with “behavioral categories”, or “traits” with “categories” (**Lines 288, 291-293, 296, 299, 631, 634, 638, 763, 788 in the revision**)

**Line 298.** The revision has replaced “evidence” with “description” (**Line 307 in the revision**)

**Line 299.** We have rephrased the sentence as “…traits we identified as displaying consistent individual differences included behaviors associated with exploration and activity…” as suggested (**Lines 308-309 in the revision**)

**Line 306.** We have rephrased the words as “a sex difference” (**Line 316 in the revision**)

**Line 316.** Harrison et al. (2022) is a meta-analysis study and concluded that the patterns in sex dependent personality across species appear insignificant or inconclusive. This conclusion and the interpretation are indicated in the text (**Lines 323-326 in the revision**). The citation of Harrison et al. (2002) meant to specify that only one study on one bat species focusing on one behavioral trait was adopted in the meta-analysis, which supports our statement that still little is known about the individual differences and between-sex behavioral tendencies in most bats (**Lines 326-328 in the revision**)

**Line 333.** The typo has been corrected as “relatively” (**Line 343 in the revision**)

**Line 347.** We have rephrased the words as “…TB context was more confined in space…” (**Line 363 in the revision**)

**Line 353.** We have rephrased the words as “…engage in their predominant and natural locomotion mode…” (**Line 369 in the revision**)

**Line 360.** We have revised the words as “individuals’ behaviors” (**Line 375 in the revision**)

**Line 368.** We have revised the words as “to be bolder” **(Line 383 in the revision)**

**Lines 220, 256, 279, 308, 667, 668, 693, 694, 749, 750.** The revision has has replaced “climbing distance” and “climbing area” with “distance climbed” and “area climbed”, respectively (**Lines 229, 266, 288, 318, 691, 692, 717, 718, 773, 774 in the revision**)

**Associate Editor in filling the position of Reviewer 2**

Thanks for the Associate Editor’s comment and offering the opportunity. This manuscript reports a study of comparing the behavioral variation and repeatability of bats across different types of behavioral assays as testing contexts in assessing the potential effects of locomotive constraint on animal personality in wild bat populations. The key significances of this study are as follows.

1. Our study provides the first description of animal personality, and suggestive evidence of behavioral syndrome, in widely caught bent-wing bats and in East Asian tropical forests. More importantly, our results indicate the limitation of the commonly used standardized hole-board box (the HB test) as a test assay on studying behavioral types of bats, and pinpoint the necessity of applying devises such as flight-tents in incorporating the predominant locomotion mode, the flight, for most bats. Specifically, for this final conclusion, we’ve offered suggestive guidelines for future studies of animal personality and behavioral variation targeting on bats as the subjects.

2. Animal personality studies are much less common in bats considering the diversity of bats, which represent a unique taxon for their morphological constraints imposed on locomotion. Thus, our study has filled the gap.

3. Our study incorporated different experimental assays that mimicked specific spatial situations encountered by the bats in real situations and associated with the locomotion modes (i.e., quadrupedal movement vs. flying) of the bats. This is also a novel approach.

4. The assays used in our study included one standardized test assay (the hole-board box), one assay that is generally for studying flying and echolocation behaviors of bats but rarely specifically for examining the repeatability (the flight-tent), and a new assay that is our own design (the tunnel box) as a modified version of the maze test and has never been used previously. By this multiple-assay design, our study demonstrated the effects of test context associated with different locomotion modes of bats on the variation and repeatability of animal personality traits of bats.
